# Clinical outcomes of a cohort of patients with central nervous system metastases from thyroid cancer

**DOI:** 10.1530/EC-16-0049

**Published:** 2016-11-30

**Authors:** Joana Simões-Pereira, Daniel Macedo, Maria João Bugalho

**Affiliations:** Endocrinology DepartmentInstituto Português de Oncologia de Lisboa, Francisco Gentil, Lisboa, Portugal

**Keywords:** thyroid cancer, central nervous system, brain metastases, brain surgery

## Abstract

**Introduction:**

Metastases to central nervous system (M_1_-CNS) are rarely reported in thyroid cancer (TC) patients. We aimed to characterize patients with M_1_-CNS from TC followed in our department.

**Methods:**

Review of the medical records of 27 patients with TC-related M_1_-CNS.

**Results:**

Mean age at TC diagnosis was 56.9 ± 19.1 years. Papillary TC (55.6%) was the commonest histological type, followed by poorly differentiated (18.5%), medullary (11.1%), follicular (7.4%) and Hürthle cell (7.4%) carcinomas. Angioinvasion and extrathyroidal extension were observed in a high number of patients. At M_1_-CNS diagnosis, other distant metastases were already present in 77.8% of the patients. Treatment directed to M_1_-CNS was offered to 20 (74%) patients: 1 was submitted to surgery, 18 to radiotherapy (either whole-brain radiotherapy or stereotaxic radiosurgery or both) and 4 to surgery and radiotherapy. Four patients received cytotoxic chemotherapy and one was submitted to ^131^I. Median survival since M_1_-CNS detection was 5.0 months. The only factor associated with better survival was surgery to brain metastases (*P* = 0.012).

**Conclusions:**

The management of these patients is very challenging given the inexistence of effective treatments, except for brain surgery in selected cases.

## Introduction

Thyroid cancer usually has a favorable prognosis with a reported 10-year survival for differentiated and medullary thyroid carcinoma of approximately 80–95% (1) and 68–85% (2, 3), respectively. Distant metastases occur in a minority (4–15%) of these patients (4, 5, 6, 7, 8), lungs being the most commonly affected organ (9). Metastases to central nervous system (CNS) are unusual, occurring in 1% of the patients with thyroid cancer (10, 11, 12). In contrast with lung involvement, CNS metastases can cause acute disabling symptoms and a marked reduction of patients’ survival. International guidelines (13) recommend surgical resection of CNS metastases regardless of its radioactive iodine (RAI) avidity. Those patients not eligible for surgery may undergo stereotaxic radiosurgery (SRS) or whole-brain radiotherapy (WBR). RAI can be considered for iodine-avid CNS metastases, under glucocorticoid therapy to minimize the TSH-induced effects and the inflammatory process related with RAI.

We aimed to characterize a series of 27 patients with CNS metastases from thyroid cancer in terms of demography, histological features of the primary tumor, diagnostic approach, treatment modalities and clinical outcomes.

## Subjects and methods

Retrospective analysis of patients referred to a single center and identified through the South Regional Cancer Register of Portugal, and the database of our department. Histological diagnosis of thyroid cancer, of any type, and CNS metastases (encephalic parenchyma involvement and/or leptomeningeal carcinomatosis) from this tumor were considered as inclusion criteria.

Patients with coexisting malignant neoplasms and/or CNS involvement due to skull bone metastases were excluded from analysis. Finally, clinical records were reviewed to evaluate the demographic characteristics, clinical presentation, diagnostic approach, therapeutic modalities and clinical outcome.

The results presented herein resulted from a retrospective study based on the analysis of clinical files, according to the rules established by the Local Ethics Committee. This study was approved by the Ethics Committee of Instituto Português de Oncologia de Lisboa, Portugal. The results presented in our manuscript resulted from a retrospective study based on the analysis of clinical files, according to the rules established by the Local Ethics Committee. In many cases, the patients had already passed away, so it was not possible to collect the consent forms in these cases.

Statistical analysis was performed using IBM SPSS Statistics, version 21. Mann–Whitney test was used to compare means, which were described as mean ± s.d. and medians were reported as median (minimum–maximum). Survival after CNS metastases was calculated with Kaplan–Meier method, and log-rank test was used for univariate analysis. A two-sided *P* < 0.05 was considered significant.

## Results

Twenty-seven patients with CNS metastases from thyroid cancer were identified, 18 (66.7%) were women. Median follow-up was 4 (3–49) years. The diagnosis of CNS metastases was established between 1984 and 2014.

### Primary thyroid cancer: age, histological findings, staging and treatment

Mean age at diagnosis was 56.9 ± 19.1 years. Twenty-six patients (96.3%) were submitted to total thyroidectomy; from these, 9 (34, 6%) were also submitted to any type of neck lymph node dissection. One (3.7%) had no thyroid surgery. Histological characteristics and staging are shown in Table 1.

**Table 1:** Histological characteristics and staging.

**Characteristics**	**Number of patients** (percentage)
Histological type	
• PTC	15 (55.6%)
Classical variant	6 (22.2%)
Follicular variant	4 (14.8%)
Macrofollicular variant	1 (3.7%)
Classical + follicular variants	1 (3.7%)
Classical + columnar variants	1 (3.7%)
Tall-cell variant	1 (3.7%)
Follicular + solid + cribiform variants	1 (3.7%)
• FTC	2 (7.4%)
• HCC	2 (7.4%)
• MTC	3 (11.1%)
• PDTC	5 (18.5%)
Multifocality	7 (25.9%)
Angioinvasion	16 (59.2%)
Extrathyroidal extension	13 (48.1%)
Resection	
R0|R1|R2|Rx	9 (33.3%)|5 (18.5%)|5 (18.5%)|8 (29.6%)
Stage at diagnosis	
• T	
pT1|pT2|pT3|pT4|pTx	4 (14.8%)|2 (7.4%)|8 (29.6%)|8 (29.6%)|5 (18.5%)
• N	
pN0|pN1a|pN1b|pNx	5 (18.5%)|3 (11.1%)|6 (22.2%)|13(48.1%)
• M	
M1|M0/x	11 (40.7%)|16 (59.3%)

All the 4 patients with tumor size ≤2 cm (pT1) presented aggressive histological characteristics: poorly differentiated areas in 2 and angioinvasion, multifocality and lymph node metastases in 3.

Further to surgery, 22 out of 24 patients (92.0%) with thyroid cancer from the follicular epithelium underwent radioactive iodine (RAI) treatment. Worth of note was a patient who was submitted to six RAI treatments (total ^131^I activity of 575.5 mCi), 5 after the diagnosis of CNS metastases (patient 8, Table 2).

**Table 2:** CNS metastases-directed therapeutic approaches.

	**Histological type**	**Gender**	**Age at CNS-M1 diagnosis**	**CNS-M1 single/multiple**	**PS**	**Other distant metastases?**	**Brain surgery** (resection)	**RT**	**Best response**	**Other treat.**	**Follow-up since CNS-M1 detection** (months)	**Status at last follow-up**
1	PTC	F	78	Single	3	No	Complete	WBR	SD		37	Alive
2	PTC	F	70	Multiple	0	Yes	–	WBR	PD		2	Alive
3	PTC	F	52	Multiple	3	Yes	Incomplete	SRS + WBR	SD	C	35	Dead
4	PTC	M	76	Multiple	3	Yes	–	WBR	PD		2	Dead
5	PTC	M	69	Single	2	Yes	Complete	–	SD		7	Dead
6	PTC	F	66	Multiple	3	Yes	–	WBR	PD		5	Dead
7	PDTC	F	81	Single	0	Yes	–	SRS	SD		25	Dead
8	PDTC	F	40	Multiple	1	Yes	–	–	SD	131I + C	51	Dead
9	FTC	F	26	Single	0	Yes	–	WBR	PD		8	Dead
10	PTC	M	50	Multiple	3	No	–	WBR	PD		3	Dead
11	MTC	F	37	Multiple	2	No	–	WBR	PD		3	Dead
12	PTC	F	84	Multiple	0	No	–	WBR	SD		25	Dead
13	PTC	F	61	Multiple	1	Yes	Incomplete	SRS + WBR	SD		76	Dead
14	PTC	F	61	Multiple	1	Yes	–	SRS + WBR	SD		18	Dead
15	HCC	M	63	Single	0	Yes	–	WBR	PD		2	Alive
16	MTC	M	33	Single	0	Yes	–	SRS + WBR	PD		2	Dead
17	PTC	F	80	Multiple	1	Yes	–	WBR	PD		4	Dead
18	PTC	F	45	Multiple	0	Yes	–	WBR	PD	C	3	Dead
19	PDTC	M	44	Single	0	Yes	–	WBR	PD	C	9	Dead
20	PTC	M	59	Single	3	Yes	Complete	WBR	PD		10	Alive

C, chemotherapy; CNS-M1, central nervous system metastases; F, female; FTC, follicular thyroid cancer; HCC, Hurthle cell carcinoma; M, male; MTC, medullary thyroid cancer; PD, progressive disease; PDTC, poorly-differentiated thyroid cancer; PS, performance status; PTC, papillary thyroid cancer; SD, stable disease; SRS, stereotaxic radiosurgery; treat, treatments; WBR, whole-brain radiotherapy.

### CNS metastases: diagnosis, therapeutic approaches and clinical outcome

Of the eleven (40.7%) patients who already had distant metastases at diagnosis, in 3, (11.1%) the CNS metastases were the first manifestation of the disease. Median interval between the diagnosis of thyroid cancer and CNS metastases was 44 (0–513) months independently of the histological type. This interval was not different between the patients with different tumor’s histological type (*P* = 0.620). Mean age at diagnosis of CNS metastases was 62.0 ± 16.3 years. Twenty-one (77.8%) patients had other distant metastases. Among these, 8 (3.1%) showed concomitantly lung and bone metastases (mainly in vertebral column and skull).

The most frequent neurological complaints, referred by 74.1% of patients, were headaches and dizziness. In the remaining cases, patients were asymptomatic, and the diagnosis was disclosed by imaging examinations during restaging.

Sixteen patients (59.3%) had a single CNS metastasis. Mean size of the dominant metastasis was 30.4 ± 15.5 mm.

CNS metastases-directed therapeutic approaches were offered to 20 patients and are summarized in Table 2. Of those 17 (62.7%) who were submitted at some point to WBR, 2 (7.4%) developed alopecia, 1 (3.7%) suffered an ischemic stroke and 1 (3.7%) cerebral demyelination.

Systemic cytotoxic chemotherapy was offered to 4 (14.8%) patients: doxorubicin + cisplatin (cases 8 and 19, Table 2), capecitabine (patient 3, Table 2) and paclitaxel (case 18, Table 2). One of these patients (case 19, Table 2) had been previously treated with tyrosine kinase inhibitors (TKIs) (7 months with sorafenib and 1 year with sunitinib) and CNS metastases developed under this treatment.

### Survival after CNS metastases’ diagnosis

At the time of last follow-up, 4 (14.8%) patients were still alive. Median survival since the diagnosis of thyroid cancer was 59 months and since CNS metastases’ detection was 5.0 months. The only factor associated with better survival was surgery to brain metastases (*P* = 0.012). Variables/conditions considered in the analysis are described in Table 3.

**Table 3:** Univariate survival analysis.

**Variables**	**Median survival in months**
Age at CNS metastases’ diagnosis (≤45 vs >45 years)	*P* = 0.745 (≤45 = 7 vs >45 = 3)
Gender	*P* = 0.558 (F = 8 vs M = 7)
Histological tumor type*	*P* = 0.375 (PTC = 7 vs HCC = 1 vs MTC 3 vs PDTC 9)
Multifocality*	*P* = 0.851 (yes = 7 vs no = 9)
Angioinvasion*	*P* = 0.976 (yes = 9 vs no = 4)
Distant metastasis at TC diagnosis	*P* = 0.814 (yes = 5 vs no = 7)
CNS metastases diagnosis (incidental vs neurological symptoms)	*P* = 0.911 (incidental = 5 vs neurological symptoms = 8)
Number of CNS metastases	*P* = 0.392 (single = 7 vs multiple = 5)
Surgery to CNS metastases	*P* = 0.012 (yes = 76 vs no = 4)
WBR	*P* = 0.259 (yes = 8 vs no = 1)
SRS	*P* = 0.177 (yes = 25 vs no = 4)

* Relative to thyroid cancer.

CNS, central nervous system; F, female; HCC, Hurthle cell carcinoma; M, male; MTC, medullary thyroid cancer; *P*, *P* value; PDTC, poorly differentiated thyroid cancer; PTC, papillary thyroid cancer; SRS, stereotaxic radiosurgery; WBR, whole-brain radiotherapy.

Those patients not treated for brain metastases (*n* = 7) had a significant lower median survival compared to those who were submitted to at least one therapy (1 month vs 8 months; *P* = 0.008). Although not statistically significant, these patients were older at brain metastases diagnosis (70.7 ± 9.0 vs 59.0 ± 17.5 years; *P* = 0.162). They evidenced a plurimetastatic disease and the majority also presented multifocal CNS lesions with disabling symptoms (confusion, vomits, convulsive crisis, etc.); they died soon after the diagnosis of CNS involvement. Furthermore, in the vast majority of patients, it was not possible to clearly establish whether the cause of death was directly dependent from the progression of CNS metastases or from the disseminated disease progression.

## Discussion

It is generally accepted that CNS metastases from thyroid cancer are rare. However, a recent systematic review by Madani and coworkers (14) revealed that, among 492 patients with differentiated thyroid cancer and rare sites of end-organ distant metastases, the brain was the most commonly affected organ within the rare locations. In 1986, McConahey and coworkers (15) verified that 15% of the patients with metastatic papillary thyroid carcinoma developed CNS metastases along their disease course. Furthermore, necropsy studies from patients who died from thyroid cancer (16, 17) have also documented a frequency of 9.3–20% of CNS metastases.

Most patients with CNS involvement presented concomitant distant metastases, mainly in lung and bones. In the present series, 77.8% of the patients had other metastases at the time of CNS metastases’ diagnosis. In other published series, this percentage was even higher (Table 4).

**Table 4:** Summary of the series reporting CNS metastases from thyroid cancer.

	***n***	**Histological type**	**Age at CNS-M1** (years)	**Other distant M1** (%)	**Surgery to CNS-M1** (%)	**SRS** (%)	**WBR** (%)	**Survival after CNS-M** (months)	**Period between thyroid cancer and CNS-M1** (months)
1. Current study	27	15 P, 2 F, 2 H, 3 M, 5 PD	62	77.80	18.50	18.50	63	5	44
2. Henriques de Figueiredo *et al*. (18)	21	12 P, F 5, 4 PD	63	85.70	47.60	9.50	71.40	7.1	32
3. Bernard *et al*. (28)	23	9 P, 2 H, 1 M; 11 unknown	63		52.20	65	34.80	20.8	41.8
4. McWilliams *et al*. (20)	16	11 P, 2 F, 1 A, 1 H, 1 M	47	87	56.30	25	62.50	17.4	77.1
5. Salvati *et al*. (29)	12	3 P, 3 F, 5 A, 1 M	51	58	75	75		19.8	37.4
6. Chiu *et al*. (19)	47 (38 pre-mortem)	32 P, 11 A 4 M	59	66	23.70	–	52.60	4.7	34.8
7. Samuel and Shah (21)	15	9 F, 5 P, 1 H	46	60	40	–	40	12.4	61.8

CNS, central nervous system; CNS-M_1_, central nervous system metastases; F, follicular; H, Hurthle-cell; M, medullary; P, papillary, PD, poorly-differentiated; SRS, stereotaxic radiosurgery; WBR, whole-brain radiotherapy.

As expected, there was a higher frequency of aggressive tumors such as Hürthle cell carcinoma (HCC), medullary thyroid carcinoma (MTC) and poorly differentiated thyroid carcinoma (PDTC) in this subpopulation of patients with CNS metastases, comparative to the frequencies observed among patients with thyroid carcinoma as a whole. However, the histological subtypes did not influence either the period of time elapsed between the initial diagnosis of thyroid cancer and evidence of CNS metastases (*P* = 0.595) or the median survival (Table 3). In our institution, PTC represents 83.7% of all thyroid tumors, follicular thyroid carcinoma (FTC) 9.4%, MTC 3.3% and PDTC 1.4% (unpublished data).

In most patients with well-differentiated thyroid cancer, angioinvasion and extrathyroidal extension coexisted with multifocality, poorly differentiated areas and/or aggressive variants, like insular, solid and tall-cell variants. The few patients lacking angioinvasion/extrathyroidal extension presented nonetheless aggressive histopathological features. On the other hand, all the patients with medullary or poorly differentiated thyroid cancer presented aggressive microscopic features.

Although MTC has a different biological behavior from follicular epithelium-derived thyroid cancer, the 3 patients with this subtype of tumor did not evidence a distinctive clinical course, except for a diagnosis of CNS metastases under forty years of age, in 2 out of 3 cases.

We compared our results with those from previous studies including at least 10 patients with CNS metastases from thyroid carcinoma (Table 4). As in other series, the most common histological type was papillary thyroid carcinoma (PTC). Worth of note is one case diagnosed with CNS metastases 42 years after the diagnosis of lung metastases that were the first manifestation of a papillary microcarcinoma (case 3, Table 2).

Median survival after diagnosis of CNS metastases is highly variable among series, probably reflecting different therapeutic approaches. In our series, median survival was less than 10 months after CNS metastases’ diagnosis, as observed in two other series (18, 19). Although not statistically significant, patients with incidentally diagnosed M_1_-CNS had a lower survival (incidental CNS diagnosis = 5 months vs patients with neurological symptoms = 8 months; *P* = 0.911). In these patients, the diagnosis was established after clinical suspicion of progression and by imaging tests performed to restaging disease. Therefore, the survival is likely to reflect the unfavorable outcome associated with disseminated disease.

As a rule, the treatment option depends on a multidisciplinary team judgment considering the general condition of the patient, the dissemination of the primary tumor, the location and the number of CNS lesions. In line with previous reports, the only factor associated with better survival was surgery to brain metastases, which in our series was performed in a small number of cases. Surgery was preferably offered to patients with good performance status (PS) and with lesions amenable for resection (unimetastases/oligometastases). All but one evidenced a single CNS metastasis. This fact, combined with a probable better performance status might have contributed to a longer survival among patients submitted to brain surgery. Therefore, at least to the eligible patients, surgery appears as an option that ameliorates the prognosis. Given that this study encompassed a long period, different technical limitations over time cannot be excluded.

Performance status was considered by Henriques de Figueiredo and coworkers (18) as an important prognostic factor in patients with brain metastases from thyroid cancer. These authors verified an overall survival of 27 months when patients’ PS was <2 and 3 months when PS ≥2 (*P* = 0.0009). Due to the retrospective nature of this study, we could not assess the performance status of patients at the time of brain metastases diagnosis, because this information was not clearly described in the vast majority of the medical files. The prognostic value of unifocality or multifocality of metastases is still not clearly established (19).

Iodine uptake by brain metastases is infrequent (up to 17% (19, 20, 21)) and, therefore, RAI therapy is not considered as a very efficient approach. However, isolated cases have been reported with satisfactory results (22), as seen in one of our patients who was submitted to 6 RAI treatments (5 after the diagnosis of CNS metastases), following preparation with recombinant thyrotropin, and who evidenced a survival of 51 months. Currently, to avoid prolonged stimulation with TSH, the use of recombinant TSH is recommended (13). RAI-related neurological symptoms have been described, including cerebral edema (23), but this risk may be mitigated with concomitant glucocorticoid therapy.

As in other studies (18, 19, 20), WBR was not a predictor of longer survival in our series. This therapeutic approach can be proposed to patients for whom surgery is contraindicated, who have disseminated or inaccessible lesions or in whom life expectancy is less than 3 months (19). However, its benefits in disease control must be weighted against its potential neurotoxicity.

Conventional cytotoxic chemotherapy did not offer clear benefits. The use of capecitabine was tried in one patient also without clinical benefit. Capecitabine is an oral prodrug of fluorouracil, previously associated with favorable responses in patients with breast cancer and brain metastases heavily pretreated with other cytostatics including 5-fluorouracil.

Targeted therapies with TKIs are now an alternative for advanced thyroid cancer. The use of these drugs (specifically Sorafenib) has been described in the setting of brain metastases secondary to thyroid cancer (24, 25). The single case that we treated with TKIs developed CNS metastases under this treatment. A major concern of this approach is the risk of CNS bleeding associated with the use of TKIs (26, 27).

The major limitations of this study are (1) its retrospective nature and (2) recruitment of patients encompassing a large period of time along which technical resources were necessarily different.

CNS metastases are rare and present a poor prognosis. Currently, there are no truly effective therapeutic approaches that can improve patients’ survival, except for brain surgery, as we observed in our patients. However, we recognize that our sample is small and it may be difficult to get suitable patients for surgery (good clinical status and single or few CNS metastases) at the time of CNS metastases’ diagnosis. Therefore, the majority of these patients only benefit of supportive care and/or inclusion in clinical trials directed at treatment of thyroid cancer brain metastases.

## Declaration of interest

The authors declare that there is no conflict of interest that could be perceived as prejudicing the impartiality of the research reported.

## Funding

This study was funded by Associação de Endocrinologia Oncológica (AEO).
